# Culling Dogs in Scenarios of Imperfect Control: Realistic Impact on the Prevalence of Canine Visceral Leishmaniasis

**DOI:** 10.1371/journal.pntd.0002355

**Published:** 2013-08-08

**Authors:** Danielle N. C. C. Costa, Cláudia T. Codeço, Moacyr A. Silva, Guilherme L. Werneck

**Affiliations:** 1 Program for Scientific Computing, Oswaldo Cruz Foundation (Fiocruz), Rio de Janeiro, Brazil; 2 School of Applied Mathematics (EMAP) of the Getulio Vargas Foundation (FGV), Rio de Janeiro, Brazil; 3 Department of Epidemiology, Social Medicine Institute, State University of Rio de Janeiro (UERJ), Rio de Janeiro, Brazil; University of Pittsburgh, United States of America

## Abstract

**Background:**

Visceral leishmaniasis belongs to the list of neglected tropical diseases and is considered a public health problem worldwide. Spatial correlation between the occurrence of the disease in humans and high rates of canine infection suggests that in the presence of the vector, canine visceral leishmaniasis is the key factor for triggering transmission to humans. Despite the control strategies implemented, such as the sacrifice of infected dogs being put down, the incidence of American visceral leishmaniasis remains high in many Latin American countries.

**Methodology/Principal Findings:**

Mathematical models were developed to describe the transmission dynamics of canine leishmaniasis and its control by culling. Using these models, imperfect control scenarios were implemented to verify the possible factors which alter the effectiveness of controlling this disease in practice.

**Conclusions/Significance:**

A long-term continuous program targeting both asymptomatic and symptomatic dogs should be effective in controlling canine leishmaniasis in areas of low to moderate transmission (R_0_ up to 1.4). However, the indiscriminate sacrifice of asymptomatic dogs with positive diagnosis may jeopardize the effectiveness of the control program, if tests with low specificity are used, increasing the chance of generating outrage in the population, and leading to lower adherence to the program. Therefore, culling must be planned accurately and implemented responsibly and never as a mechanical measure in large scale. In areas with higher transmission, culling alone is not an effective control strategy.

## Author Summary

Visceral leishmaniasis is listed as a neglected tropical disease and is considered a public health problem worldwide. The disease has been documented since 1885, the first case being reported in India. After over 120 years, the incidence of the disease remains high despite control strategies implemented. In areas where the disease is zoonotic, such as in Brazil, identification as well as removal of infected dogs is recommended in highly endemic areas for they are considered to be the reservoir of the *Leishmania chagasi* parasite. The theoretical basis that supports the culling of infected dogs is the assumption that the incidence of human infection is directly related to the number of infectious dogs. However, there is no consensus among researchers on the effectiveness of this strategy for controlling either human or canine visceral leishmaniasis. In this context, mathematical models can provide a basis for determining the strategies with the greatest potential for success. This paper aims to contribute to this discussion by introducing further complexities into the problem, in particular, the imperfect diagnosis of this infection and the time gap between laboratory diagnosis and culling and the presence of asymptomatic infections.

## Introduction

Visceral leishmaniasis or kala-azar is the most severe clinical form of leishmaniasis, a serious public health problem worldwide [1], [2]. In Latin America, the agent of the so-called American visceral leishmaniasis (AVL) is *Leishmania (Leishmania) chagasi* transmitted, in Brazil, mainly by sandfly *Lutzomyia longipalpis* [Lutz & Neiva, 1912]. So far, the findings related to the epidemiology of AVL point to a spatial correlation between the occurrence of disease in humans and high rates of infection in dogs, suggesting that, in the presence of the vector, canine visceral leishmaniasis is a key factor for triggering transmission to humans [3]. Overall, the incidence of AVL remains high despite the large-scale control strategies that have been implemented. These strategies focus on early diagnosis and treatment of human cases, vector control to reduce sandfly population, as well as the removal of infected dogs and health education [4].

Despite the lack of solid evidence in literature, culling dogs with canine visceral leishmaniasis (CVL) has been the major strategy for controlling this disease in Brazil. Many authors argue that this strategy has low cost-benefit and many are against it, often encouraging the non-delivery of animals to slaughter [5], [6], [7], [8], [9]. Other professionals, however, admit that this strategy can produce positive results [6], [10], [11]. Two possible factors associated with the low effectiveness of culling programs are: (1) the discontinuity of these programs, which may occur for several reasons, including the lack of a structured surveillance system, budget issues and lack of adequately trained professionals; (2) Problems related to the logistics in delivering control measures, for example, low infected dog screening rates and lack of a reliable and valid diagnostic test to detect dogs in the early stages of infection, leaving out asymptomatic infectious dogs that are capable of conveying the parasite to the vectors, thus, cooperating with the continuity of the transmission cycle [12], [13].

Mathematical modeling has been applied in studies of visceral leishmaniasis in order to understand the transmission dynamics of this infection [8], [14], [15], [16], [17], [18], [19] and the impact of control strategies. Hasibeder *et al.* (1992) and Dye *et al.* (1992) proposed and implemented models to estimate the basic reproduction number (R_0_) of CVL, which was estimated between 1.44 and 11, this large uncertainty being attributed to the poor performance of the available diagnostic tests. Their model predicts that in areas where R_0_ is at the upper limit of the R_0_ range, culling would be successful only if intensively implemented. In real settings, however, R_0_ estimation is highly uncertain as it depends on how the seropositivity is measured, and on the many assumptions of the underlying model, such as the homogeneous exposure of dogs to sandflies and homogeneous response to infection. Later, Dye (1996) [20] alerted that culled dogs tend to be rapidly substituted by younger and susceptible ones, reducing the effectiveness of this strategy, compared to alternatives such as vector control, drugs and vaccines.

Other studies have explored the effectiveness of imperfect control programs, assessing the effect of imperfect diagnostic tests 24, and discontinued dog culling programs [15]. They found that a high sensitivity test, together with the immediate sacrifice, was sufficient to control the disease. On the other hand, with a low sensitivity test, the effectiveness of the program was lost, whether or not the dogs were sacrificed immediately.

This paper seeks to revisit this problem, focusing on the relevance of asymptomatic infections in a scenario of imperfect control characterized by sub-optimal screening, diagnosis and slaughter rates. We further investigated an unexplored component that is the impact of the low specificity of the diagnostic test. We hope to contribute to the understanding of CVL transmission dynamics and the factors that modulate the control effectiveness.

## Materials and Methods

A mathematical model of transmission dynamics of CVL was developed and implemented in the R software, version 2.13.0 using the library deSolve [21]. The SEI_2_D model assumes that all dogs are born susceptible and the dog population abundance is constant over time (N = 10000 dogs), that there is homogeneity in the exposure of susceptible dogs to the infectious agent, implicit, sandfly mediation. The transmission coefficient is constant (**β**) and implying that the ability of the vector to transmit the parasite to dogs is constant over time. Two levels of transmission were chosen to represent areas where CVL endemicity is either low (prevalence of 3% at steady state) or high (prevalence of 12% at steady state) [22], [23]. Values of **β** under these two scenarios were obtained by finetuning this parameter to obtain the desired steady-state prevalence. The parameters are listed and defined in Table 1.

**Table 1:** Definition of symbols and corresponding values used in the model SEI_2_D.

Symbol	Meaning	Value (unit)	Reference
**S**	Susceptible	**-**	**-**
**E**	Latent	**-**	**-**
**I**	Infected	**-**	**-**
**Is**	Symptomatic Infectious	**-**	**-**
**Ia**	Asymptomatic Infectious	**-**	**-**
**μ**	Natural Mortality Rate	0,00694 month^−1^	Personal Communication
**α**	Mortality Rate Induced by infection	0,07333 month^−1^	Pozio *et al*, 1981
**λ**	Relapse Rate of “Ia” to “Is”	1/48 month^−1^	Pozio *et al*, 1981
**i**	Incubation Rate	0.319 month^−1^	Courteney, 2002
**β**	Transmission Coefficient	variable	Adopted Value
**p**	Proportion becoming asymptomatic	<0.5	Lanotte *et al*, 1979
**qa**	Infectivity of “Ia”	0.21	Courteney, 2002
**qb**	Infectivity of “Is”	0.62	Courteney, 2002
**r**	Screening rate	4.1–8.3 month^−1^	Adopted Value
**d**	Sensitivity of the Diagnostic test	0.8–1	Adopted Value
**d_z_**	1-Specificity of the diagnostic test	0–0.2	Adopted Value
**f**	Proportion sent to culling	0.25	Adopted Value
**1/u**	Delay between screening and elimination	0–4 months	Adopted Value
**s**	Dummy indicating Culling of Ia dogs	0 ou 1	Adopted Value

**Figure 1:**
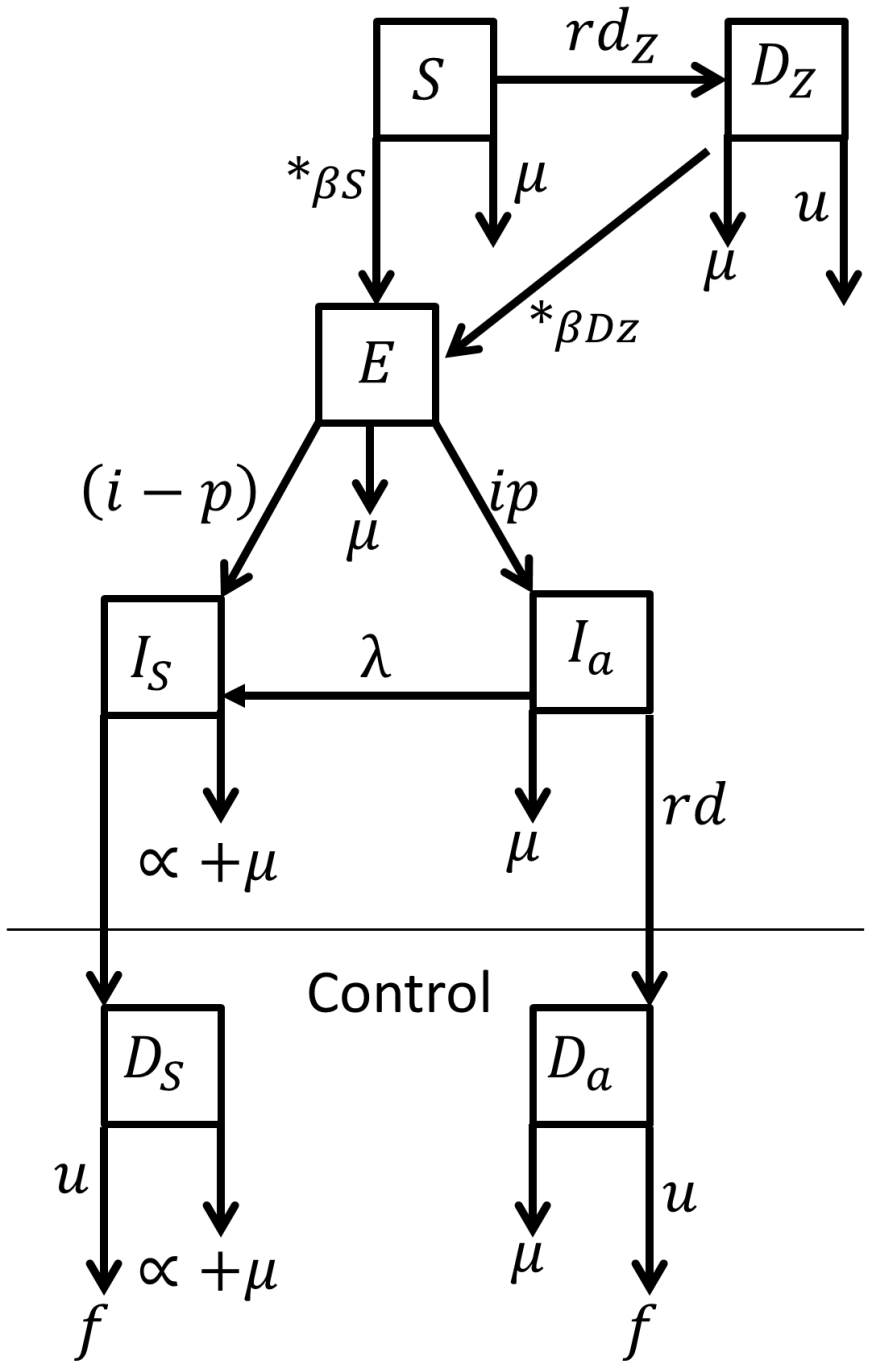
The Canine Leishmaniasis model (SEI_2_D). All dogs are born susceptible (S), and become infected at a rate **ßS**. Infected dogs go through a latent stage, after which a fraction evolves to an asymptomatic infection (Ia) while the remaining (1-p) evolve into the symptomatic state (Is). A small fraction of asymptomatic dogs may evolve to present signs of clinical disease, which is incorporated into the model through a relapse rate “λ”. The control program screens animals and, if laboratory positive, they move to class Da or Ds, where they remain until culling. Dz class holds those erroneously classified as positive.

As in Dye (1996) this model has a latent stage (E), in which recently infected dog does not manifest the disease neither transmits the parasite. However, differently from other models, here, a fraction **p** of the latent dogs evolves into an asymptomatic stage which is detectable by the diagnostic test, and contributes to the transmission at a lower rate than the symptomatic group, as suggested by the results obtained by Courtenay *et al* (2002). The remaining dogs evolve to the symptomatic syndrome. We further assume that asymptomatic dogs may eventually show signs of clinical disease, which is incorporated into the model as a small flow of individuals from the asymptomatic to the symptomatic compartment. The model has two further compartments, Da and Ds, to hold asymptomatic and symptomatic individuals respectively, correctly diagnosed as infected, and a compartment Dz for the susceptible individuals that were erroneously diagnosed as infected. Individuals in Dz, while waiting for the culling, may eventually become infected. In this case, they move from the Dz to the E compartment (Figure 1).

The SEI_2_D's equations are as follows:
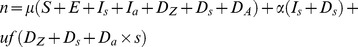
(1)

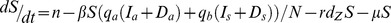
(2)

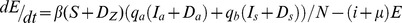
(3)

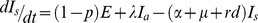
(4)

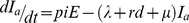
(5)

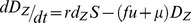
(6)

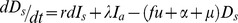
(7)

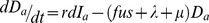
(8)


### Reproduction number

An expression for the basic reproduction number of CVL was derived from the SEI_2_D model, without control, using the next generation matrix method [24]. The mathematical derivation is found in the appendix (Text S1).

The Basic Reproduction Number is:
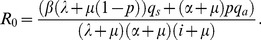
(9)


### Simulating imperfect control programs

For modeling purposes, the intervention program was divided into three components: screening, diagnosis and sacrifice. Screening measures the monthly capture rate and application of the diagnostic test to dogs in the population.
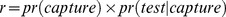
(10)


The parameter *d* represents the probability of a dog to be positively diagnosed given it has been subjected to a diagnostic test and is infected (test sensitivity).
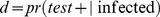
(11)


Once positively diagnosed, the dog has a chance **f** of being put down. The delay between the screening and the sacrifice is **1/u**.

The product **r x d_z_** measures the rate of misclassification of uninfected dogs. This rate depends on **r** (screening rate) and **d_z_** which corresponds to the test's probability of false positive (1- specificity).

By varying these parameters, **r, d, d_z_, u** e **f**, one can investigate the impact of a variety of imperfect control programs.

Here, we considered variations of two hypothetical programs, both of which have been continuously implemented for 40 years. The first one was based on data from the CVL control program implemented in Belo Horizonte, Brazil, considered to be of good quality, within the possibilities of the country (data provided by the Subcoordenation of Vector Transmitted Zoonosis and Rabies/SVS/MS). In this program, the screening rate is 6% per month followed by the immediate sacrifice of 85% of the screened dogs with positive diagnosis. We implemented this scenario, assuming a diagnostic test with 90% sensitivity and 100% specificity.

A second scenario was built representing a worse situation, in which the screening rate is 4% per month and time to culling is 4 months as in Courtenay *et al*. (2002). In this scenario, diagnostic tests were applied with sensitivity and specificity ranging from 80% to 100%. In both programs, we investigate two protocols: one targeting exclusively symptomatic dogs and screening all dogs, regardless of the presence or absence of symptoms.

To investigate the impact of diagnostic tests with low specificity, we compared the number of erroneously culled dogs by programs using tests with specificity of 80, 90 and 100%. By quantifying the number of dogs that were needlessly put down, we have a measure of the negative impact of the control strategy.

### Measurement of effectiveness

The effectiveness of the control programs was assessed by comparing the prevalence before and after 40 years of the establishment of the Control Program. The control program was considered successful if it were capable of reducing CVL prevalence below 1%. Considering that prevalence is measured by imperfect diagnostic methods, we further distinguished between real success and perceived success. Real success is achieved when the true prevalence decreases below 1%, while perceived success is achieved when the measured prevalence decreases below this threshold.

### Uncertainty and sensitivity analyzes

At last, to investigate the impact of uncertainties in the specification of model parameters in the success of the control programs, we performed a multivariate uncertainty and sensitivity analysis. The procedure was as follows: First, uniform probability density functions were defined for each life-history parameter (**i, qa, qb, p, λ, a, μ**) with intervals equal to 0.75 and 1.25 times the default parameter value. Secondly, one thousand values were draw from each of these distributions, producing 1000 sets of parameters. To maintain the transmission constant, for each new set of parameters, **β** was calculated from the R_0_ equation so that the R_0_ of all the simulations was kept at the same level. After running the model SEI_2_D with each set of parameters, we recorded the success of the control program after 40 years as positive if final prevalence was less than 1% and failure otherwise.

## Results

### The basic reproduction number

Using the expression of R_0_ derived from the SEI_2_D model and the parameter values presented in Table 1, we obtained R_0_ = 1.09 for the low endemicity scenario and R_0_ = 1.29 for the high endemicity scenario. These values are low compared with those reported by other authors but were based on prevalence observed in the field [21], [22]. In the sensitivity analysis section, we discuss scenarios with higher R_0_.

### Effectiveness of the realistically good control program

According to the SEI_2_D model, a CVL control program with 6% monthly screening rate, a diagnostic test with 90% sensitivity, and no delay between screening and culling should be effective in controlling CVL if implemented continuously for 40 years, that is, prevalence is reduced below 1%. In the low endemicity area, the success is reached by only targeting symptomatic dogs. Under slightly higher transmission, however, successful control requires the sacrifice of symptomatic and asymptomatic dogs. That is, limiting the intervention to clinically positive dogs was not sufficient to control the disease below the 1% prevalence level, leaving it at 2% instead.

### Effectiveness of worse control programs

**Figure 2:**
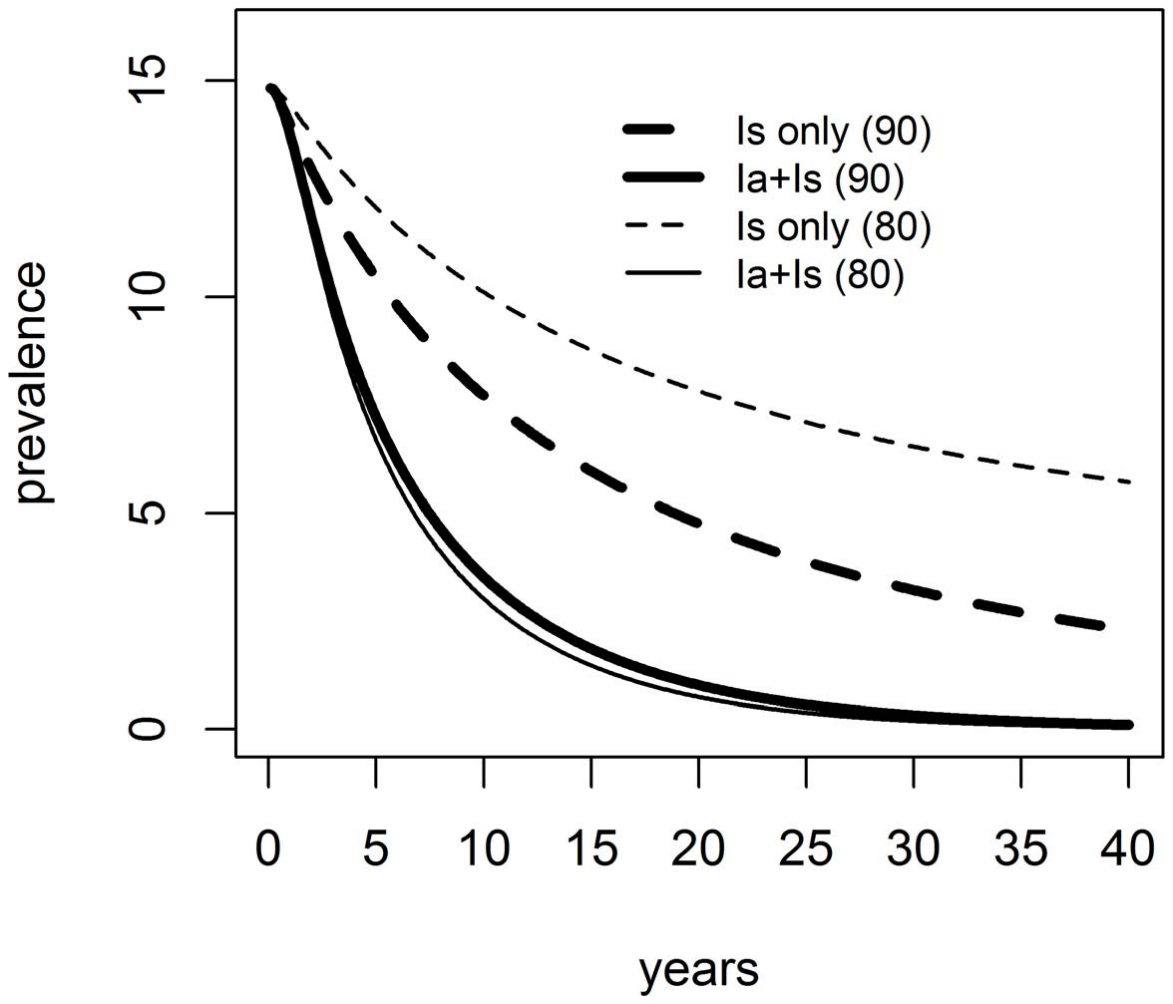
Prevalence of CVL in the higher endemicity area under four control programs differing in their target and in diagnostic test used. “Is only” = control program targeting only symptomatic dogs, “Ia + Is” = targeting asymptomatic and symptomatic dogs; the number between parenthesis indicates the sensitivity of the diagnostic test.

When screening is reduced to 4% per month, a less sensitive test is used (80%) and elimination time increases to an average of four months, the good performance of the control program targeting symptomatic dogs only is still preserved in the low endemicity area, with final prevalence reaching values below 1%. As transmission increases, targeting just symptomatic dogs becomes no longer effective, resulting in final prevalence of 6%. To ensure prevalence below 1%, at least 30% of the asymptomatic dogs should be put down (results not shown). Figure 2 shows that, if control targets both symptomatic and asymptomatic dogs, the impact of improving the sensitivity from 80 to 90% is negligible. On the other hand, in a program targeting symptomatic dogs only, improving the test's sensitivity to 90% is very advantageous to improve its effectiveness.

To further investigate the relevance of asymptomatic dogs on control, we parameterized the model once again, assuming that all asymptomatic dogs were non-infectious, but still positive for the diagnostic tests. These individuals are the dogs considered cured according to Lanotte *et al*. (1979) [25]. In this case, their elimination has no effect on the success of the control program.

The low and moderate endemicity scenarios simulated here are in the low range of the estimated values for R_0_. The performance of imperfect culling programs in an area with extremely high transmission rate, corresponding to R_0_ = 9, was evaluated and, in this case, none of the culling strategies were effective (Table 2). Actually, the R_0_ threshold under which CVL is controlled is R_0_ = 1.41 for programs targeting any seropositive dog. A maximum R_0 = _1.106 is required for the success of programs targeting clinically positive dogs only.

**Table 2:** Results of effective control of CVL with lower value of R_0_.

Control strategy		Prevalence of CVL			
Diagnostic test (%)	Target	R_0_ = 1.09	R_0_ = 1.29	R_0_ = 1.41	R_0_ = 9
E = 100 S = 80	All	0.007	0.084	1.51	71.059
	Is	1.463	5.61	16.24	77.408
E = 90 S = 80	All	0.002	0.031	0.61	70.203
	Is	0.818	3.40	12.29	76.710
E = 80 S = 80	All	0.001	0.012	0.24	69.347
	Is	0.472	2.03	8.83	76.013
E = 80 S = 90	All	0.000	0.006	0.13	68.611
	Is	0.427	1.845	8.28	75.853
E = 90 S = 90	All	0.001	0.015	0.34	69.487
	Is	0.740	3.11	11.67	76.556

Prevalence of canine visceral leishmaniasis after 40 years of a culling program featuring 4% screening rate. Observe how the success is affected by the underlying transmission rate (R_0_), the target (Is = symptomatic dogs only, Ia + Is = symptomatic and asymptomatic dogs), and the diagnostic test's specificity and sensitivity.

### Simulating the effect of a diagnostic test with low specificity

One of the main arguments against culling programs is the unnecessary sacrifice of healthy dogs that are erroneously diagnosed, leading to speeches against culling, which reduces the number of animals delivered to zoonosis centers, increasing the ethical and social costs of this strategy. Here, we calculated the number of unnecessarily sacrificed dogs in a program using a diagnostic test with 80% sensitivity and either 80% and 90% specificity, during the five years of the control application (years 35 to 40 after control implementation). In the high endemicity area, a program using a test with 80% specificity, this number was 5821 animals, which corresponds to 38% of all dogs put down. Increasing specificity to 90%, only a slight reduction was obtained. However, restricting culling to symptomatic dogs only is not sufficient to control the disease below the 1% prevalence level. This result poses a dilemma to control programs in high endemicity areas as the success of culling is only achieved if asymptomatic dogs are included and this is done at the expense of putting down non-infected dogs. In areas with low endemicity, on the other hand, restricting culling to symptomatic dogs can both control the disease and reduce the risk of putting down healthy animals.

### Uncertainty and sensitivity analysis

**Figure 3:**
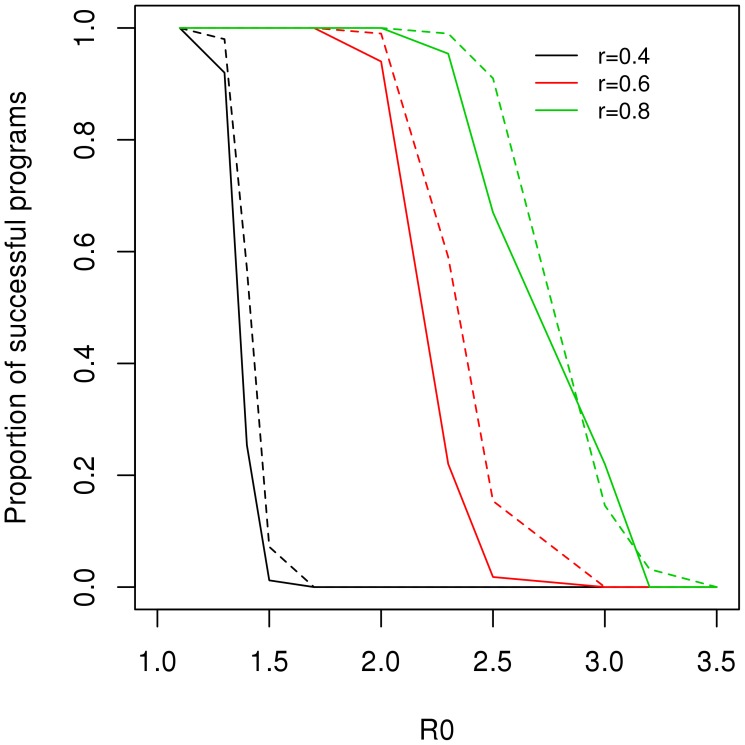
Success of culling programs as a function of the basic reproduction number (R0), the screening effort (r) and the sensitivity of the diagnostic test (solid line = 80%; dotted line = 90%).

Figure 3 shows the proportion of the parameter space – corresponding to a variety of natural history situations – that were controlled by culling programs with screening rates equal to 4, 6 or 8%, and test's sensitivity equal to 80 or 90%. It is clear that the success of the culling programs is highly dependent on the transmission rate and that increasing screening effort is required in areas with high transmission. Moreover, it is clear that increasing screening effort is more effective than increasing the sensitivity of the diagnostic tests from 80 to 90%. However, one must consider the costs associated with such effort for a routine program.

**Figure 4:**
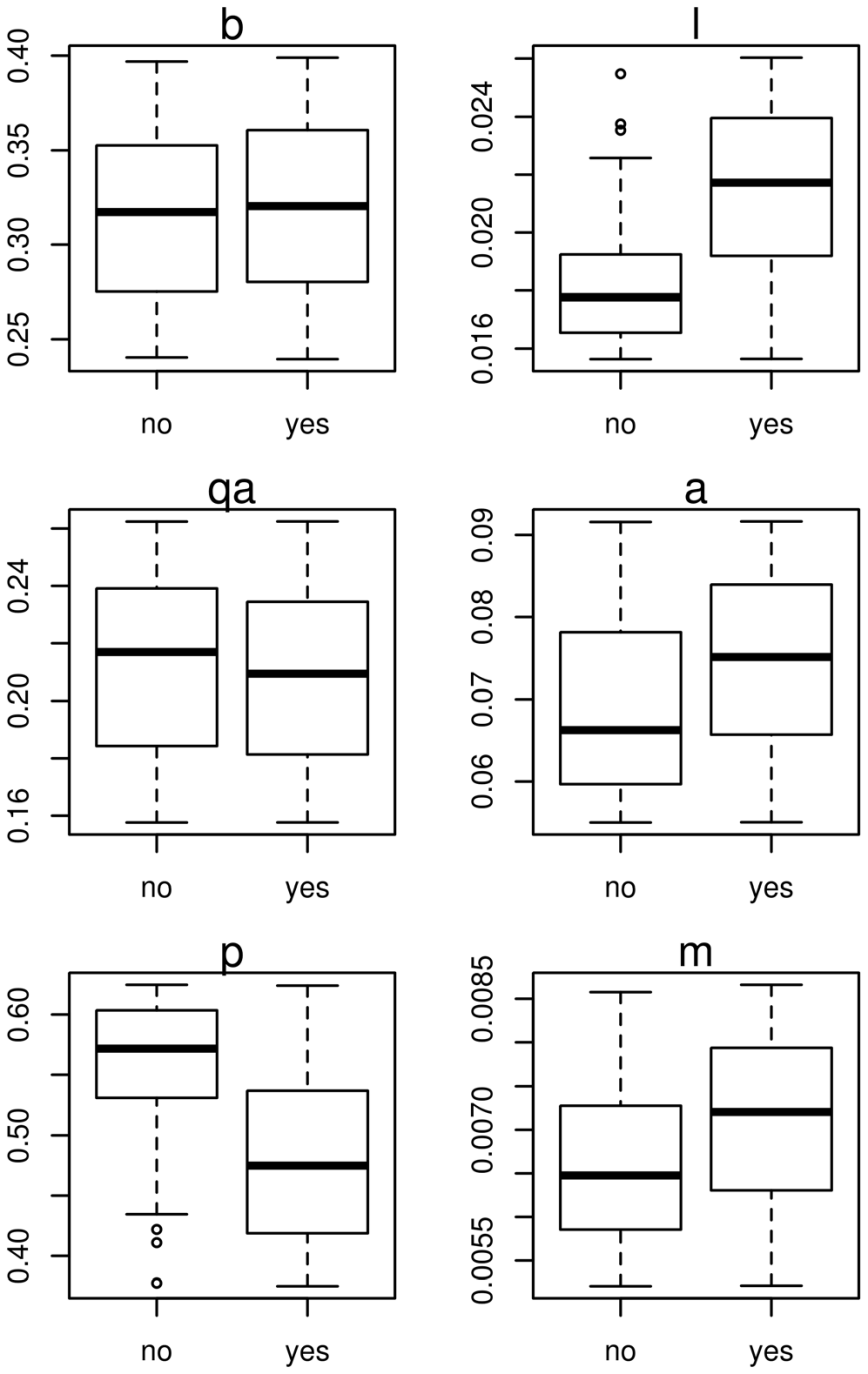
Sensitivity analysis of parameters in Model SEI_2_D. Box plots comparing parameter values that lead to prediction of successful control (prevalence <1) and failure (prevalence >1) considering a culling program with 4% screening effort and a diagnostic test with 80% sensitivity applied in an area with R_0_ = 1.41.

Figure 4 shows the life-history parameters associated with the success or failure of the control program that targeted asymptomatic and symptomatic dogs, with 0.04% screening effort in an area with R_0_ = 1.41. The most important parameters refer to the asymptomatic population. In summary, the higher the proportion of dogs becoming or remaining asymptomatic, the most effective the program is. The reason is the lower transmissibility of these asymptomatic dogs.

## Discussion

This study aimed at assessing the effectiveness of culling dogs in the control of canine visceral leishmaniasis in scenarios where implementation occurs imperfectly. This investigation was based on a mathematical model for CVL that introduces a class of infectious asymptomatic dogs which contributes, at a lower rate, to the transmission cycle [15]. This model differs from previous models, in which asymptomatic dogs are assumed to be uninfectious [7], which may be true for European dogs that are well nourished [26] but not necessarily for all dog populations. The infectiousness and proportion of asymptomatic dogs had strong impact on the success of control strategies.

As a matter of comparison, we also simulated a simple SI model as parameterized for CVL. This is in line with part of CVL modeling literature using SIR-like models [16], [27]. Overall, when compared with the SEI_2_D model, SID generates more optimistic expectations, with successful control being reached at faster rates.

Most researchers agree that the sensitivity, specificity and reproducibility of the available serological tests are substandard [6], [11], [15], [16], [25], [28], [29], [30]. Sensitivity depends on the methodology used, and the specificity varies with the choice of the antigen. Low sensitivity increases the chance of permanence of false-negative animals in the environment [12]. An aggravating issue in the permanence of asymptomatic dogs is the difficulty of tracking these dogs, turning them into a silent reservoir [12].

The main result of our simulations is that, in areas with very low transmission (baseline prevalence of 3%), culling of symptomatic dogs by a realistic program with 4% screening and testing per month and a mean delay to culling of four months, is sufficient to maintain prevalence under 1%, which we considered a successful endpoint. The advantage of this program is the focus on symptomatic dogs only, what reduces the burden of killing apparently healthy dogs, providing a better grip of the program by the population. However, the control program was successful in interrupting transmission of CVL in areas of low transmission, possibly because the endemic equilibrium in these simulations was fragile, and a simple disturbance in the system lead to R_0_<1.

In areas with slightly higher endemicity (R_0_ = 1.29, prevalence = 15%), on the other hand, removing clinically diagnosed dogs is not sufficient as a control strategy because the asymptomatic population is large enough to maintain transmission. This is in accordance with many studies [12], [13], [15], [31]. In this case, a program would have to be capable of including at least 30% of the asymptomatic but infectious dog population in order to maintain infection prevalence below 1%.

A further complication of targeting asymptomatic dogs is the increased chance of putting down healthy dogs as the diagnostic tests available have low specificity. This is a serious problem in areas with lower endemicity, where the positive predictive values of the tests tend to be low. The models studied here suggest that in the simulated area, 79% of dogs would be wrongly eliminated by tests with 80% specificity. The unnecessary sacrifice of non-infected dogs burdens the program and feeds the discourse against dog culling and increases society's aversion to the control program [12].The emotional onus and social cost of euthanizing dogs, whether they are ill or not, must be considered in evaluating of culling dogs as a control strategy against AVL. To avoid the erroneous sacrifice of false-positive dogs, it must be ensured that the tests have high specificity reducing the social cost of this strategy.

The transmission rate of CVL in real settings can be much higher than the ones simulated here [17], [20]. As the transmission rate increases, the effectiveness of the culling program rapidly declines unless investment in screening is enhanced (Figure 3). In high transmission areas, the required effort may become too high to be feasible, and combined strategies, such as vector control, may become necessary. In any scenario, control effectiveness requires continuity, that is, no interruptions in the application of control measures.

In practical terms, the inclusion of asymptomatic dogs in a control program stumbles in several difficulties: the difficulty of screening and testing these dogs, as well as their diagnosis, and convincing the delivery of apparently healthy dogs for culling [12], [31]. An excellent program would be the one which is more efficient and less costly. A control program aimed only at symptomatic dogs has apparently lower cost than one targeting all infected dogs. However, such program by itself will not control transmission.

These results overall, suggest that strategies should differ in areas with high and low transmission, with more integrated approaches being the choice in the former and culling of symptomatic dogs being a choice in the latter. We did not investigate the relative effectiveness of other strategies in the same scenario. Dye (1996) suggested that insecticide application can be more effective than culling, but this is based on the assumption that the impact of insecticides on the sandfly population is high, and resistance is absent or low.

Palatnik-de-Sousa *et al*. (2004) argues that using diagnostic tests with greater sensitivity collaborates for the greater effectiveness of a culling program, by minimizing the percentage of false-negative dogs. However, Dye *et al.* (1993) assures that even if a highly efficient serological test was used, about 20% of the cases would remain undetected, especially in animals which, at the time of the test, were in the incubation or seroconversion phases. Using tests with greater sensitivity and lower specificity may incur in greater social cost and reduced efficiency due to low social acceptance.

In summary, the analysis of the models suggests that besides investments on the improvement of diagnostic tests, further effort is required to improve the control program itself, considering the logistics and resources required for implementation of control for longer periods.

The results of this study are limited to cases in which the model is valid. The model assumed a canine population of constant size, but it is possible that, in some contexts, these populations are actually increasing or decreasing. Another limitation of the model is not explicitly considering the dynamics of the vector. It is known from the study of other diseases such as dengue and malaria that the vectorial capacity can be affected by climate and environmental conditions, including variation from one year to another. The model also does not consider other potential hosts such as wild animals. The impact of control would be lower if these animals were present. Furthermore, the model assumes that all dogs are homogeneously exposed to the risk of vector contact. In real situations, the risk is expected to vary spatially and future studies should consider this dimension. Finally, control is implemented continuously, but in real situations this is rare. Future studies should investigate the impact of strategies applied in pulses at different times of the year. With all this, we must evaluate the results of this study with caution and by a realistic point of view, noting that the canine sacrifice was effective in controlling the CVL only in a scenario in which the control was implemented monthly and with the same effort for 40 years.

## Supporting Information

Text S1 **The description of next generation matrix and R_0._** The calculation of basic reproduction number of canine visceral leishmaniase was derived from the SEI_2_D model, without control, using the next generation matrix method. The next generation matrix was calculation by mathematical derivation.
(DOC)
