# Supplementary material for: Culling Dogs in Scenarios of Imperfect Control: Realistic Impact on the Prevalence of Canine Visceral Leishmaniasis
Source: PLoS Negl Trop Dis. 2013 Aug 8;7(8):e2355. doi: 10.1371/journal.pntd.0002355 (PMC3738479; doi:10.1371/journal.pntd.0002355)
Supplement: Text S1 — The description of next generation matrix and R0. The calculation of basic reproduction number of canine visceral leishmaniase was derived from the SEI2D model, without control, using the next generation matrix method. The next generation matrix was calculation by mathematical derivation. (DOC) [file pntd.0002355.s001.doc]

**Supporting Text S1** [The next generation matrix and R_0_ calculation]

Rewriting the equations:

The next generation matrix K is built from matrices T (transmission, that is, generation of new infectious cases) and S (transfer between compartments)^10^. The T matrix refers to the appearance of new cases and the S matrix refers to transfers between infected compartments in the model. Only the infected and infectious compartments are relevant, E, Ia and Is.

Matrix T is such that. Which is $T=\left[\begin{matrix} 0 \\ 0 \\ 0 \end{matrix}\right.\begin{matrix} \\ \\ \end{matrix}\begin{matrix} \beta qa \\ 0 \\ 0 \end{matrix}\begin{matrix} \\ \\ \end{matrix}\left.\begin{matrix} \beta qb \\ 0 \\ 0 \end{matrix}\right]$.

Matrix S is given by . Which is $S=\left[\begin{matrix} -\left(i+\mu\right) \\ ip \\ i\left(1-p\right) \end{matrix}\right.\begin{matrix} \\ \\ \end{matrix}\begin{matrix} 0 \\ -\left(\lambda+\mu\right) \\ \lambda\end{matrix}\begin{matrix} \\ \\ \end{matrix}\left.\begin{matrix} 0 \\ 0 \\ -\left(\alpha+\mu\right) \end{matrix}\right]$

The next generation matrix is .

$$K=\left(\begin{matrix} \frac{\left(\beta_{i}\left(\lambda+\mu\left(1-p\right)\right)q_{s}+\left(\alpha+\mu\right)pq_{a}\right)}{\left(\lambda+\mu\right)\left(\alpha+\mu\right)\left(i+\mu\right)} \\ 0 \\ 0 \end{matrix}\right.\begin{matrix} \\ \\ \end{matrix}\begin{matrix} \\ \\ \end{matrix}\begin{matrix} \frac{\beta\left(\lambda q_{s}+\alpha q_{a}+\mu q_{a}\right)}{\left(\lambda+\mu\right)\left(\alpha+\mu\right)} \\ 0 \\ 0 \end{matrix}\begin{matrix} \\ \\ \end{matrix}\begin{matrix} \\ \\ \end{matrix}\left.\begin{matrix} \frac{\beta q_{s}}{\left(\alpha+\mu\right)} \\ 0 \\ 0 \end{matrix}\right)$$

R_0_ is the largest eigenvalue of this matrix, which in this case, is simply the element K_1;1_.

$$R_{0}=\frac{\left(\beta\left(\lambda+\mu\left(1-p\right)\right)q_{s}+\left(\alpha+\mu\right)pq_{a}\right)}{\left(\lambda+\mu\right)\left(\alpha+\mu\right)\left(i+\mu\right)}$$

Using the following values:

- β = 0.141
- qa = 0.21
- qb = 0.62
- i = 0.319148
- λ = 0.020833
- pa =0.4
- ps =0.6
- μ = 0.00694
- α = 0.07333

The next generation matrix is $K=\left[\begin{matrix} 1.37 \\ 0 \\ 0 \end{matrix}\right.\begin{matrix} \\ \\ \end{matrix}\begin{matrix} 1.88 \\ 0 \\ 0 \end{matrix}\begin{matrix} \\ \\ \end{matrix}\left.\begin{matrix} 1.08 \\ 0 \\ 0 \end{matrix}\right]$ and R_o_ = 1.3767
